# Huge Antrochoanal Polyp: A Case Report

**DOI:** 10.7759/cureus.45592

**Published:** 2023-09-20

**Authors:** Salmah M Alharbi, Anas M Alshehri, Ibrahem H Erwe

**Affiliations:** 1 Otorhinolaryngology-Head and Neck Surgery Department, Aseer Central Hospital, Abha, SAU; 2 Otorhinolaryngology-Head and Neck Surgery Department, Khamis Mushayt General Hospital, Khamis Mushayt, SAU

**Keywords:** maxillary sinus, huge, choking, case report, antrochoanal polyp

## Abstract

Antrochoanal polyps are benign lesions arising from the maxillary sinus mucosa. The most common patient complaint is unilateral nasal obstruction. Nasal endoscopy and computed tomography are the gold standard modalities for diagnosis. Treatment is surgical. We report the case of a 19-year-old female suffering from a huge antrochoanal polyp who went to hospital following a choking episode. While being prepared for surgery, she vomited, and the polyp was expelled from her mouth. Right maxillary sinus antrostomy was done to remove the polyp base to avoid recurrence.

## Introduction

An antrochoanal polyp (ACP), also known as Killian's polyp, is a benign lesion originating from the maxillary sinus, protruding from the natural or accessory ostium into the middle meatus, then passing posteriorly to the choana and nasopharynx. The exact pathogenesis of ACP is unknown; however, its relationship with allergic rhinitis or ipsilateral maxillary sinusitis has been shown in pediatric patients [1]. In addition, several theories have been hypothesized to explain the pathogenesis of ACP, including its growth from an antral mucous retention cyst [2] or herniation through the accessory ostium due to increased intra-sinus pressure from partial occlusion of the natural ostium caused by inflammatory changes and edema [3]. Diagnosis is mainly clinical, aided by radiological imaging. The most common presentation is unilateral nasal obstruction. Other reported symptoms include dysphagia, speech disturbances, and foreign body sensation in the throat [4]. Autoamputation of the polyp with nasal or oral expulsion is rarely reported [5-7].

Nasal endoscopy and computed tomography are the gold-standard diagnostic modalities [4]. The currently recommended treatment is surgical removal of the polyp through functional endoscopic sinus surgery. The chosen surgical procedure should provide the widest exposure helping the identification of the polyp origin [8]. Here, we report a case of a large ACP presenting with choking. While preparing for surgery, the polyp was expelled orally during a vomiting attack.

## Case presentation

A 17-year-old Saudi female was referred at around 9:30 a.m. to our hospital after suffering from a choking episode at 5 a.m. the same day. The patient complained of foreign body sensation in the throat. There was no history of respiratory distress, cyanosis, or a previous choking episode. Before that day, she complained of nasal obstruction but did not have any other symptoms related to the nose. She was diagnosed earlier with inferior turbinate hypertrophy. There was no complaint of drooling. There was no history of fever or recent infection.

On examination, the patient was vitally stable with an oxygen saturation of 96% in room air. She had nasal speech, and throat examination revealed a huge non-pulsating antrochoanal polyp extending to the epiglottis inducing gag reflex. Bilateral inferior turbinate hypertrophy was seen in the anterior nasal examination. Computed tomography of the paranasal sinuses was urgently requested to rule out other anomalies. It showed a large mass in the right maxillary sinus extending to the right nasal cavity and descending to the nasopharynx and oropharynx down to the epiglottis, markedly narrowing the airways. Frontal, sphenoidal, and ethmoidal sinuses were clear. There was no air-fluid level, and the bony boundaries of the paranasal sinuses were intact. The diagnosis was right maxillary ACP.

**Figure 1:**
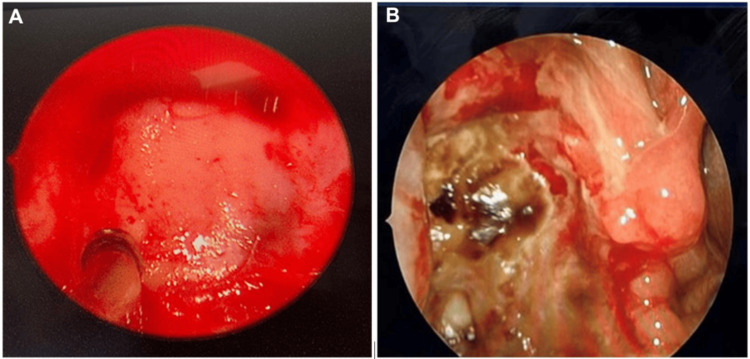
A. Intraoperative photo showing right maxillary sinus wide antrostomy with the base of the polyp seen, removed, and cauterized. B. Postoperative follow-up photo showing right maxillary sinus antrostomy with no recurrence, only crustation and clots that were cleaned.

The patient was admitted, and emergency functional endoscopic sinus surgery was scheduled. While the patient was waiting for the operation, she had a severe gag reflex and vomiting, ending with oral expulsion of the polyp without complications. She was rushed to the operating room where she underwent septoplasty, right maxillary sinus antrostomy, and removal of the polyp origin and base (Figure 1A). The patient was discharged home the next day on nasal spray and oral antibiotics. Postoperative follow-up showed improvement with no signs of recurrence (Figure 1B).

## Discussion

We report a unique case of a 19-year-old female who had a huge ACP and presented with a foreign body sensation and a choking episode. Oral expulsion of the polyp occurred during vomiting. Although the most common presentation of ACP is unilateral nasal obstruction, our patient was used to experiencing nasal blockage due to a history of inferior turbinate hypertrophy. This could explain why she only started complaining after the polyp grew and caused airway narrowing. It is worth mentioning that anatomic variations, including inferior turbinate hypertrophy, were studied as an etiology of ACP. Frosini et al., in a large case series on 200 cases with ACP, reported that anatomical variations, such as nasal septal deviation, concha bullosa, and accessory ostium, were factors with significant correlation [1,4]. Baser et al. concluded that anatomic variation stood out as an important factor in ACP development [9]. One theory suggests anatomical variations can cause increased pressure in the maxillary sinus [10]. Hammouda et al. referred to inferior turbinate hypertrophy among pathologies associated with ACP in their case series [11].

Nasal obstruction is the most common presenting symptom, and it has been noted in almost all patients with ACP [12]. However, ACP may cause a wide variation of clinical manifestations, including rhinorrhea, snoring, headache, mouth breathing, epistaxis, anosmia, dyspnea, dysphagia, and dysphonia. These presentations may have some similarity to other nasal disorders, such as mucocele, angiofibroma, hemangioma, and malignant tumors of the nasopharynx [3,13,14]. Anterior rhinoscopy usually reveals an intranasal polypoidal mass and confirms the diagnosis [4].

The oral expulsion of ACP reported in our case is a rare occurrence. Large polyps with long stalks are prone to torsion and ischemia due to passing through narrow ostia. This can lead to infarction and expulsion following a sudden increase in nasal pressure as in sneezing or vomiting. There are few reported cases of nasal or oral ACP expulsion [5-7].

Although expulsion of the polyp already occurred in our case, the patient underwent endoscopic surgery for septoplasty and right maxillary antrostomy. Simple polyp avulsion is associated with a high incidence of recurrence [15]. Recent literature suggests that the key to preventing recurrence is the accurate identification and removal of the origin of the polyps. Endoscopic sinus surgery with middle meatal antrostomy using angled endoscopes proved to be both effective and safe, with the advantages of reduced morbidity, operating time, and duration of hospital stay [11,16].

## Conclusions

We report a case of a huge antrochoanal polyp reaching the level of epiglottis. The polyp was autoamputated and expelled from the mouth during vomiting. Septoplasty, right maxillary sinus antrostomy, and removal of the polyp origin were performed to prevent recurrence.
